# Delayed elimination communication on the prevalence of children's bladder and bowel dysfunction

**DOI:** 10.1038/s41598-021-91704-3

**Published:** 2021-06-11

**Authors:** Peng Chao Xu, Yi He Wang, Qing Jun Meng, Yi Bo Wen, Jing Yang, Xi Zheng Wang, Yan Chen, Yu Lin He, Qing Wei Wang, Yan Wang, Lin Gang Cui, Jennifer D. Sihoe, Israel Franco, Jing He Lang, Jian Guo Wen

**Affiliations:** 1 Pediatric Urodynamic Center and Department of Urology, First Affiliated Hospital of Zhengzhou University, Zhengzhou, 450052 China; 2 Henan Joint International Pediatric Urodynamic Laboratory, First Affiliated Hospital of Zhengzhou University, Zhengzhou, 450052 China; 3 Department of Urology, Section of Pediatric Urology, Yale University, New Haven, CT 06520 USA; 4 Department of Obstetrics and Gynecology, Peking Union Medical College Hospital, Beijing, 100005 China

**Keywords:** Bladder disease, Neurogenic bladder, Urinary incontinence, Urinary tract infection, Urinary tract obstruction, Risk factors, Disease prevention, Health policy, Paediatrics, Gastrointestinal diseases

## Abstract

To determine the prevalence of bladder and bowel dysfunction (BBD) and its relationship with delayed elimination communication (EC) in children. A cross-sectional study was carried out in kindergartens and primary schools in mainland China. A total of 10,166 children ranging from 4 to 10 years old were included. A total of 10,166 valid questionnaires were collected, and 409 children were diagnosed with BBD. The overall prevalence was 4.02% (409/10,166) and decreased with age, from 6.19% at age 4 to 1.96% at age 10. With the prolonged use of disposable diapers (DDs), the commencement of usage of EC in a child was significantly put off or delayed by parents, and the prevalence of BBD amongst these children increased (*P* < 0.001). The prevalence of BBD among children who stopped using DDs within the first 12 months and after more than 24 months was 2.79% and 4.38% respectively. Additionally, the prevalence among children who started EC within 12 months after birth and those who never engaged in EC was 1.36% and 15.71% respectively. Early introduction of EC and weaning of DD usage has a positive correlation with lower prevalence of BBD in children in China.

## Introduction

Bladder and bowel dysfunction (BBD) is highly prevalent worldwide and is thought to result from the interplay of multiple factors that vary regionally. The coexistence of voiding dysfunction symptoms and functional constipation and/or faecal incontinence (FI) in children was previously termed ‘dysfunctional elimination syndrome’ (DES)^1,2,3^. However, there was no standardized definition of DES for children until 2013 when the International Children’s Continence Society (ICCS) suggested the term ‘bladder and bowel dysfunction’ instead of DES as a descriptive comprehensive term of a combined bladder and bowel disturbance that does not explain pathogenesis but rather encompasses this parallel dysfunction. Lower urinary tract(LUT) dysfunctions common to BBD include storage symptomps like urinary frequency, urgency, incontinence, nocturia, as well as voiding symptomps like hesitancy, dysuria and other issues like over active bladder and underactive bladder, meanwhile, defecation complaints accompanied by the LUT dysfunctions are like constipation, functional non-retentive faecal incontinence(FNRFI) and/or encopresis^2,3^. BBD can predispose children to urinary tract infection (UTI), urinary incontinence and other complications such as vesicoureteral reflux (VUR)^4^. Not only is it therefore a difficult and distressing problem for children and their families but can impose a sense of guilt and embarrassment amongst them with some children ending up as victims of bullying^1,5^.

In some children with neuropathic diseases, such as congenital spina bifida, neural tube defects and congenital megacolon, a clear common neurological basis is identifiable. A functional interaction between the bladder and bowel has been postulated, due to their close anatomical proximity, and both share innervation of the parasympathetic S_2_-S_4_ and sympathetic L_1_–L_3_ nerve roots^2^. However, no neurological basis for the combined dysfunction of the two organs has been obviously recognized.

Elimination communication (EC) is also known as ‘natural infant hygiene’ and sometimes referred to as ‘baby-led potty training’ or ‘assisted infant toilet training’. Elimination refers to the act of defecation or urination. Elimination communication is a two-way process in toilet training. When the child shows cues of elimination, such as crying, squirming, straining, wriggling, grimacing, fussing and vocalizing, the caregiver can coordinate this elimination process with audio cues (soft whistle or hum) whilst holding or sitting the child with thighs apart over the toilet to complete this process rather than to allow them to eliminate in their DDs^5^.

The timing of toilet training may vary somewhat between countries and cultures. It has been suggested that children less than two years old are not considered ready for toilet training because of immature bladder and bowel control as well as a lack in physical skills to go to a toilet and remove their own clothes by themselves. The American Academy of Pediatrics (AAP) had suggested that children should use DDs until they are considered ready for toilet training^6^. However, whether DDs should be used before and until the maturation of voiding and defecation skills remains debatable. Furthermore, whether early active defecation guidance and discontinued DD usage combined with commencement of EC and potty training can effectively decrease the occurrence of BBD in children remains to be answered.

**Figure 1:**
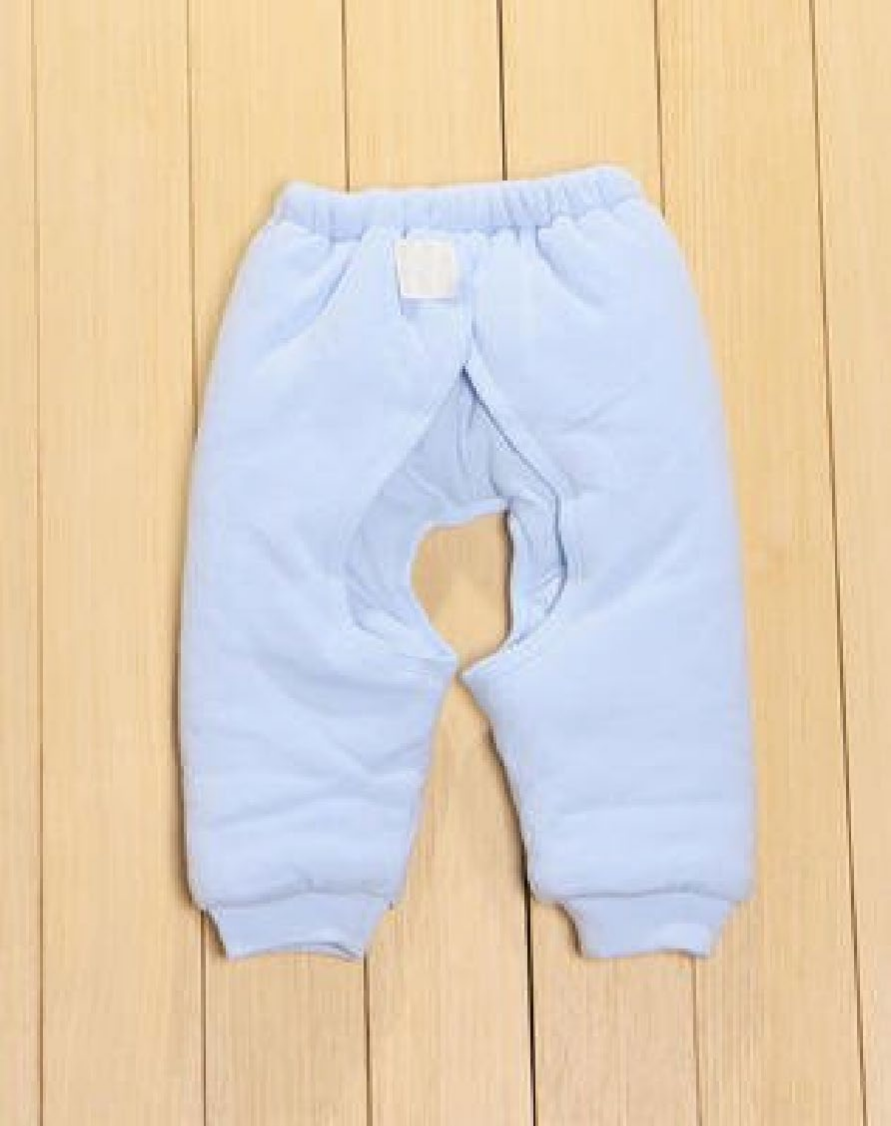
With split-crotch pants, it is more convenient for parents to wean off diapers in a timely manner and to allow the children to control their own urination and defecation. (Photo taken and provided by the first author and the colleagues).

**Figure 2:**
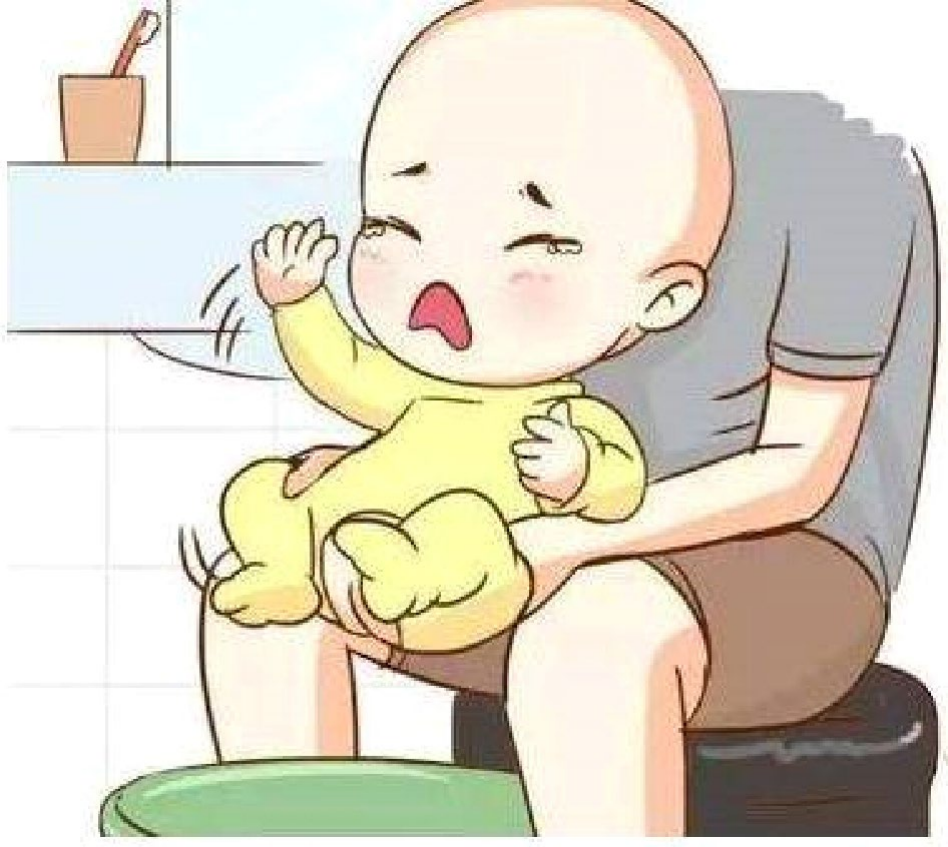
Caregivers can coordinate EC with holding out and separating the thighs (‘Baniao’ in Chinese). (Photo drawn and provided by the first author and the colleagues).

Traditionally, in China, many families may just use a cloth or split-crotch pants (Fig. 1) instead of DD or after weaning off DDs. They also practice “Baniao” (a Chinese term) which describes lifting of the child in a semi-squatting position with their thighs apart over the toilet or potty (Fig. 2). Some of the intrinsic connection may should be discussed in more detail, particularly the internal relationship between DD use and delayed EC or delayed toilet training, as well as the relationship between BBD prevalence and EC start and end times. DD use cessation was the period of cessation of diaper use at the discretion of the caregiver proactively, from assisted infant toilet training gradually to the start of toilet training independently. Bladder control was considered achieved when the child is aware of the need to void, able to express the need with verbal and/or non-verbal communication, managed to stay dry and have no urinary retention^7^.

Although the physiological mechanisms of BBD have been previously examined in a number of studies, no definite conclusions have been drawn. It has also been reported that excessive dependence on DDs and subsequent delayed EC may be associated with BBD symptoms, such as voiding frequency and/or urgency, an unstable bladder, incontinence and other defecation problems^8^. It is therefore worth exploring, whether voiding and defecation problems in young children may be related to prolonged usage of DD and delayed EC, particularly before the age of 2 years. Epidemiological surveys of BBD prevalence and its risk factors can help elucidate the pathogenesis.

This study used a questionnaire to investigate the voiding and defecation conditions of 11,285 children in different regions of mainland China. The purpose of this study was to determine the relationship between the prevalence of BBD, delayed EC and DD usage.

## Methods

### General information and methods

From March 2018 to June 2019, an epidemiological survey was performed in 12 cities with high population densities located throughout mainland China: Shenzhen (south), Xiamen (southeast), Zhengzhou (central), Xi'an (west) and Harbin (north). Nineteen kindergartens and 18 primary schools in total were selected by means of systematic sampling; classes were randomly chosen according to the children’s age distributions in the kindergartens/primary schools.

The investigation was approved by the Research Ethics Committee of the First Affiliated Hospital of Zhengzhou University and informed consent forms were obtained from all patients or legal guardian for participation in this study, including agreement of publication of identifying information or images in an online open-access publication without name,gender,age and other personal private information. Before the survey, medical staff engaged in questionnaire collection and guidance to fill in the questionnaires were trained to avoid the respondents’ misunderstanding and uncertain recall of the time of EC and DD use. As derived from parents or caregivers filling in the validated BBD questionnaire, the number of children surveyed per school was more than 200. The cross-sectional paper survey consisted of a self-administered anonymous questionnaire completed by the parents and/or caregivers. If some parents were unsure about some questions, taking the questionnaires home to complete them and returning them within one week was allowed. The procedure for filling out the questionnaires and the BBD diagnostic criteria were in accordance with the ICCS guidelines^3^.

The main contents of the questionnaire included the following: ① General information: sex, age, date of birth, height, weight, details of primary caregivers (including parents, grandparents and babysitters); ② DD usage (whether DDs were used; age when DD use was stopped; whether DDs were used during the day, at night, or all day; and adverse reactions); ③ the start time of EC and the onset of toilet independence; ④ the current voiding and defecation behaviours; ⑤ start time of urination training/bowel training, time of urination/defecation independence. The information and contents of the questionnaires were kept confidential and only known by medical staff participating in this study.

### Inclusion, exclusion and diagnostic criteria

The inclusion criteria were as follows: children aged 4 to 10 years old with normal urinary anatomy and those who had not experienced surgery for the urinary system, pelvic organs, or nervous system. BBD was defined according to the 2013 ICCS guidelines, which describe a combination of functional bladder and bowel disturbances. Accordingly, any cases who meet this definition and conform with the information collected by questionnaires will be classified as BBD group. The specific types of voiding and defecation abnormalities associated with BBD were diagnosed according to the International Classification of Diseases 10th Revision (ICD-10) and Diagnostic and Statistical Manual of Mental Disorders 5 (DSM-V), and the functional defecation disorder part of BBD spectrum was based on the Rome III criteria^2,3^. In the kindergartens and primary schools we surveyed, all children have a routine physical examination once or twice a year, including routine urine, abdominal and urinary ultrasonography, so according to the results of a prior medical examination, the exclusion criteria were as follows: children who have organic diseases, taking medication for, or had surgery of the urinary, gastrointestinal and/or nervous system. Children with current UTIs were also excluded from the study because concurrent symptoms such as urgency, voiding frequency and urge incontinence may be related to the UTI, resulting in interference or false positives in our diagnosis of BBD.

### Statistical analysis and processing

Statistical analysis was performed using the Statistical Package for Social Sciences (SPSS), version 11.0 for Windows (IBM Corp., Armonk, NY). The quantitative data with a normal distribution are expressed as the ${\overline{\text{x}}}$ ± s. Fisher’s exact tests and χ^2^ tests were used for group distribution comparisons, and the multigroup rates were compared using χ^2^ tests, trending χ^2^ tests and logistic regression.

## Results

### Questionnaire collection

A total of 11,050 questionnaires were distributed, and 10,166 valid questionnaires were included in the analysis, comprising 5118 (50.34%) males and 5048 (49.66%) females. The response rate for the questionnaires was 92% (10,166/11,050).

### The prevalence of BBD and its associations with different genders and ages are shown in Table 1 and Fig. 3

**Table 1:** Prevalence of BBD in children of different ages from 4 to 10 years old.

Age	Male			Female			Total			χ^2^	*P*
	n	BBD (%)	95%	n	BBD (%)	95%	n	BBD(%)	95%		
4	720	41 (5.69)	4.00–7.39%	686	46 (6.71)	4.83–8.58%	1406	87 (6.19)	4.93–7.45%	0.619	0.432
5	756	40 (5.29)	3.69–6.89%	745	44 (5.91)	4.21–7.60%	1501	84 (5.60)	4.38–6.69%	0.269	0.604
6	773	37 (4.79)	3.28–6.29%	755	38 (5.03)	3.47–6.60%	1528	75 (4.91)	3.83–6.01%	0.050	0.823
7	726	22 (3.03)	1.78–4.28%	725	30 (4.14)	2.68–5.59%	1451	52 (3.58)	2.63–4.54%	1.288	0.256
8	728	21 (2.88)	1.67–4.10%	733	24 (3.27)	1.98–4.57%	1461	45 (3.08)	2.19–3.97%	0.186	0.667
9	747	18 (2.41)	1.31–3.51%	744	22 (2.96)	1.74–4.18%	1491	40 (2.68)	1.86–3.50%	0.428	0.513
10	668	12 (1.80)	0.08–2.81%	660	14 (2.13)	1.02–3.22%	1328	26 (1.96)	1.21–3.70%	0.182	0.669
Total	5118	191 (3.73)	3.21–4.25%	5048	218 (4.32)	3.76–4.88%	10,166	409 (4.02)	3.64–4.41%	2.265	0.132

*P < 0.05 was considered statistically significant.

**Figure 3:**
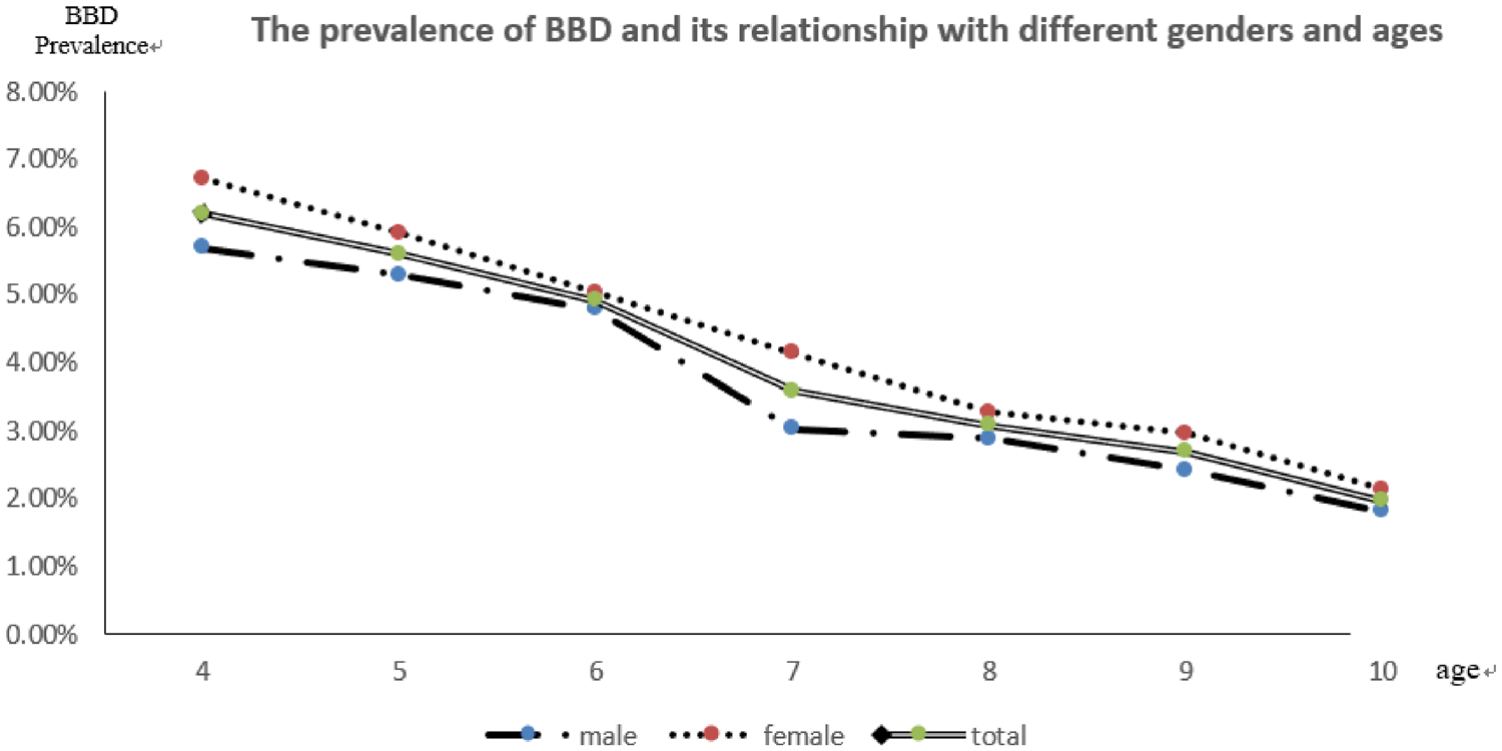
The prevalence of BBD according to gender and age.

As shown in Fig. 3, the prevalence of BBD decreased with age (*P* < 0.001). The average BBD prevalence was 3.73% (191/5,118) in boys and 4.32% (218/5,048) in girls. No significant difference in prevalence of BBD between boys and girls was noted (*P* > 0.05).

### The prevalence of BBD was related to the length of DD usage and the EC starting time (Table 2)

**Table 2:** Relationship between the prevalence of BBD and DD usage and EC.

Related factor	n	BBD (%)	χ^2^	*P*	P_sub_			
Period of DD usage/Time of weaning of DD (age in months)					a	b	c	d
No DD used	1045	11(1.05)	69.428	< 0.001		0.001	< 0.001	< 0.001
0–12	2756	77(2.79)					0.001	< 0.001
13–24	4500	197(4.38)						< 0.001
25 +	1865	124(6.65)						
**Time of DD use**								
Never	1045	11(1.05)				< 0.001	0.101	< 0.001
Only during daytime	1074	54(5.03)					< 0.001	0.368
Only at night	2962	53(1.79)	103.005	< 0.001				< 0.001
The entire day	5085	291(5.72)						
**Starting time of EC (age in months)**								
Never	643	101(15.71)				< 0.001	< 0.001	0.194
0–12	4870	66(1.36)					< 0.001	< 0.001
13–24	3906	143(3.66)	483.25	< 0.001				< 0.001
25 +	747	99(13.25)						

*P < 0.05 was considered statistically significant for every two subgroups.

*To verify whether there were differences among the subgroups, we further conducted comparisons between each of them. The letter ‘a’ represents the first subgroup, including those who never used DDs/never engaged in EC; the letter ‘b’ represents the second subgroup, including those who used DDs for less than 12 months, used DDs only in the daytime or started EC within 12 months after birth; the letter ‘c’ represents the third subgroup, including those who used DDs for 13–24 months, used DDs only at night or started EC at 13–24 months after birth; and the letter ‘d’ represents the fourth subgroup, including those who used DDs for over 25 months, used DDs for the entire day or started EC after 24 months of age.

Results showed: ① According to the Spearman correlation coefficient analysis (r = 0.236 and *P* < 0.001), with prolonged usage of DDs, there was a significant delay in starting EC and significantly increased the prevalence of BBD(*P* < 0.001). ② The prevalence of BBD was significantly lower amongst children who never used DDs (*P* < 0.001) as compared to those children who used DDs until 12 or 24 months of age. However, BBD prevalence was also significantly lower amongst children who stopped DD usage before 12 months when compared to those who used DD until after 24 months (*P* < 0.001). The BBD prevalence was also higher amongst children who used DDs during the daytime or for the entire day compared with those that never used DDs (*P* < 0.001). Moreover, children using DDs during the day are more likely to have BBD (*P* < 0.001) when compared with children who only use DDs at night. ③ With regards to the introduction of EC, prevalence of BBD was lower amongst children who started EC within 12 months of age compared with those who started EC after 12 or 24 months (*P* < 0.001).

### Risk and protective factors for BBD prevalence

To screen out the main influencing factors affecting the prevalence of BBD, the following factors were simultaneously introduced into the logistic regression model for analysis (Table 3).

**Table 3:** Logistic regression analysis of factors influencing BBD prevalence.

	$\beta$	SE	Wals	*P*	*OR*	*OR* 95% *CI*	
						Lower limit	Upper limit
**Period of DD usage/Time of weaning of DD (age in months)**							
Never			4.941	0.176	1 (ref)		
0–12	0.709	0.339	4.386	0.036	2.033	1.047	3.949
13–24	0.709	0.334	4.522	0.033	2.033	1.057	3.909
> 25	0.745	0.340	4.810	0.028	2.107	1.082	4.100
**Time of DD use**							
Never			55.650	< 0.001	1 (ref)		
Only during daytime	0.546	0.173	9.967	.002	1.727	1.230	2.424
Only at night	− 0.941	0.159	34.934	< 0.001	0.390	0.286	0.533
**Starting time of EC (age in months)**							
Never			279.834	< 0.001	1 (ref)		
0–12	− 2.643	0.185	203.922	< 0.001	0.071	0.050	0.102
13–24	− 1.713	0.145	139.919	< 0.001	0.180	0.136	0.240
> 25	− 0.404	0.167	5.839	0.016	0.668	0.481	0.927

*P < 0.05 was considered statistically significant compared with the reference.

The results of logistic regression analysis(Table 3) showed: ① The continuous prolonged use of DDs (for over 12 and 24 months) and daytime usage of DDs are risk factors for BBD in children (OR > 1, *P* < 0.05); ② DD usage only at night seemed to be a protective factor against BBD (OR < 1, *P* < 0.05); and ③ starting EC within 12 or 24 months of age was a protective factor against BBD(OR < 1, *P* < 0.05).

## Discussion

Disposable diapers are undoubtedly one of the greatest inventions of the twentieth century and is widely used around the world. In China, it has become increasingly popular over the past few decades. With their strong water absorption ability, children using DDs can sometimes go a whole day without needing to use the toilet. Based on this strong absorption ability and also disposable features, DDs are now also used for some Chinese medical workers who need to work long hours to combat COVID-19 and sometimes amongst astronauts.

It is well known that the prevalence of BBD gradually decreases with increasing age due to the natural maturation of the body. However, with the popularisation of DD usage, it is not known whether this has had any effect on the prevalence of BBD. To our knowledge, there is very limited literature addressing this and our study is one of very few studies involving such a large study population addressing issues of DD usage, EC and/or prevalence of BBD^7,9^. This kind of study is made possible in China as DD usage only became popularised in recent decades and there are still many families who have not yet resorted to DD usage. Therefore, although this is not a multicentre study, the population base provides good comparison for DD and non-DD usage. Also, as culturally most families in China had been using the technique of early EC in toilet training before the introduction of DDs, and there seemed to be a shift towards postponed introduction of EC in children on DDs, it is also interesting to see whether this has any effect on toilet training in children and, in this study, on the prevalence of BBD. Our results, using the Spearman correlation coefficient analysis (r = 0.236, *P* < 0.001) had clearly demonstrated the interaction between the prolonged use of DDs and delayed EC. Logistic regression showed that the prolonged use of DDs during the daytime is a risk factor for BBD (OR > 1, *P* < 0.05). As children usually only engage in EC training during the daytime, DD usage during the day meant less time practicing EC and may have a negative impact. This was also supported by the finding that DD usage only at night and not day was a protective factor for BBD. Furthermore, starting EC within 24 months of age was a protective factor for BBD and even more so if before 12 months of age.

Elimination Communication is not actually a new concept. It is means of assisted infant toilet training, especially useful for little children who can’t walk to the toilet by themselves, and is described as the process in which caregivers assist and enable children to meet their basic cleanliness and health needs for toileting from early infancy via verbal and nonverbal communication, or the initiation of potty use regardless of its frequency^7^. The specific implementation method of EC can involve placing the child in a certain posture to help urination or defecation, such as thighs up and apart, buttocks down, leaning against the adult abdomen, and then allowing the baby to urinate in the toilet or in the urinal above (as in Fig. 2). Whilst doing this, parents or caregivers may issue a verbal cue such as a "hush" sound until the baby urinates or defecates. There has been documentation of practice of EC dating back centuries and even in more recent years, in most resource-limited regions where DD is not widely and easily availability or affordable. However, where DD is readily available, it has quickly become popular and widely used due to its strong water absorption capability and disposable convenience, and has become a necessity for solving voiding and defecation problems during childcare^10,11^. The popularisation of DD usage is not without its downsides, such as its impact on the environment as well as other social/family issues. In China, children are often left under the care of caregivers such as grandparents whilst parents are at work during the day. Due to the convenience of DDs, caregivers (as well as parents) have a tendency for prolonged use of the DD and thus delaying the introduction of EC and toilet training. It is postulated that overlooking early EC and/or assisted infant toilet training may decrease opportunities for increasing the bladder's normal urine storage capacity and urethral and anal sphincter tension through everyday behaviours as well as remove a training method by which children can gradually develop self-controlled voiding and defecation behaviours^11,12^. However, the opinions regarding EC start time are not consistent between different countries and regions. In China, for children who do not use DDs, can sometimes start as early as 1 to 2 months of age. Caregivers are able to observe whether a baby needs to defecate based on the baby's facial, vocal, and physical features or expression. The aim is to minimize contamination of the child's body by their urine or faeces and to reduce the cleaning of cloth diapers.

In the 2003 edition of the AAP guidelines for children's toilet training, it is recommended that training should be started when the infant’s nerves, muscles, language and bladder sphincter can be controlled, usually between 18 months and 4 years of age^6^. More recent articles have challenged this as it has been suggested that an effective period for cultivating spontaneous voiding and defecation from a physiological point of view should be around 6 months of age^5,13^. At age 6 months of age or older, if the caregiver responds to the baby's excretion request, communication between the caregiver and the infant can be established, and this is referred to as ‘auxiliary baby stool training’ or EC^14,15^. Although children cannot fully learn to void and defecate spontaneously before age 2 years, the nerve reflex or biological rhythm formed in the drainage tract can cultivate the habit of spontaneous voiding and defecation. The normal functions of urine storage, voiding and defecation are dominated by sympathetic, parasympathetic and somatic nerves, and they are ultimately coordinated by the spinal cord, brainstem, midbrain and higher cortical structural pathways^16^. Beginning in the neonatal period, the functions of the bladder and bowel are regulated by nerve pathways connected to the cerebral cortex, while infancy voiding and defecation control functions are gradually cultivated through acquired learning^17^, including EC.

It is known that the internal and external urethral sphincters (EUS) are vital for urinary control. The internal urethral sphincter functions as a unit with the trigone and bladder base to store urine and is controlled by the sympathetic nervous system via the hypogastric nerve (T_10_–L_2_), while the EUS and skeletal muscles are controlled by parasympathetic and somatic motor neurons via the pudendal nerve (S_2_–S_4_)^18,19^. The EUS is comprised of inner smooth muscle surrounded by outer skeletal muscle, which contains both slow- and fast-twitch fibres, with the slow-twitch fibres being more important than the fast-twitch fibres for maintaining tonic force in the urethra. Contraction of the EUS, coaptation of the mucosa, and engorgement of blood vessels in the lamina propria contribute to voiding continence^20^.

Studies have shown that more than 17% of children have long-term urinary tract symptoms at school age, and 0.7–29.6% of children have constipation and/or FI^21,22^, which is defined as the excretion of stools in places inappropriate in the social context at least once per month in children with a developmental age of 4 years or older. Healthy children accommodate a rectal balloon with a volume of only 20 ml before they have a sudden urge to defecate, while children who have problems with chronic constipation can accommodate a rectal balloon with a volume up to 120 ml before they feel the sensation to defecate, indicating that many of the children have retention of stool in the rectal vault. One way to explain faecal retention is illustrated by Siggaard as the ‘iceberg phenomenon’, whereby only a small portion of the iceberg is visible floating above the surface and the majority is submerged^23^. In approximately 95% of children with FI, no organic cause can be identified, resulting in a diagnosis of functional defecation disorder^24^. The 80% of children with functional FI have the symptom associated with functional constipation (FC) with faecal impaction causing overflow incontinence, which is characterized by the involuntary loss of soft stools when passing through obstructing faecal mass^25^. In the remaining 20% of children with functional FI, there are no signs of faecal retention, classified as FNRFI^26^.

In addition to the proven influential factor of delayed EC, the potentially possible causes of BBD may be functional voiding and defecation disorders, arousal disorders from sleeping, mental and psychological issues, hereditary tendency, endocrine dyscrasia and hormonal instability, different methods of raising children, and different living and growing environments^8,28^. In order to facilitate the filling of the questionnaires and avoid recall bias as much as possible, the schools of the respondents we selected were mainly distributed in cities and towns, and therefore, it is unable to analyze the results between children in the schools of the urban and rural areas in this study. Meanwhile, the limitations like incorrect memory of the duration of the use of DD and the age the children start EC might occur in the present study, although we have tried our best to avoid recall bias during the survey and the later data analysis, which we will strive to improve in the future research. From our current study, EC can have a preventive role against BBD, and seems to have a role in reducing the progression of BBD even when introduced a little later in toilet training and may thus even have a therapeutic role in BBD management. The bladder and rectum are closely related in anatomy and function. Approximately 20–50% of children with bowel dysfunction have combined bladder symptoms, demonstrating that voiding and defecation function can affect each other. One study by Luise Borch analysed 73 children in Denmark and demonstrated that the standardized treatment regimen of BBD led to the resolution of functional defecation disorder in 70 of the 73 children (96%). Of the children with DUI, 68% had at least a 50% reduction in the number of daytime incontinence episodes attributable to the successful relief of bowel dysfunction, and 27% achieved complete continence during the daytime^2,27^. Another study found that while actively treating intestinal problems, 95% of the children experienced improved bowel function, and 68% of the children experienced simultaneous reduced urinary incontinence during the day, with some of the children's enuresis symptoms even disappearing. At the same time, the treatment of voiding dysfunction can also reduce the prevalence of constipation and FI among children^13,29^.

## Conclusions

The prevalence of BBD has increased significantly during recent decades in mainland China, where DDs have exhibited a much increased utilization rate in recent years. The present study showed that prolonged DD usage (particularly during the daytime) and resulting delayed introduction of EC in toilet training, are significant risk factors for BBD in our population of children. Early introduction of EC and weaning of DD usage, is associated with a lower prevalence of BBD in children.

## Abbreviations

BBD: Bladder and bowel dysfunction
EC: Elimination communication
DD: Disposable diaper
